# Development of amplified fragment length polymorphism (AFLP) markers for the identification of Cholistani cattle

**DOI:** 10.5194/aab-61-387-2018

**Published:** 2018-10-12

**Authors:** Muhammad Haseeb Malik, Muhammad Moaeen-ud-Din, Ghulam Bilal, Abdul Ghaffar, Raja Danish Muner, Ghazala Kaukab Raja, Waqas Ahmad Khan

**Affiliations:** 1 National Center for Livestock Breeding, Genetics & Genomics, PMAS Arid Agriculture University, Rawalpindi, 46300, Pakistan; 2 Department of Animal Breeding & Genetics, Faculty of Veterinary & Animal Sciences, PMAS Arid Agriculture University, Rawalpindi, 46300, Pakistan; 3 Animal Science Institute, National Agriculture Research Council, Islamabad, Pakistan; 4 Department of Biotechnology, Faculty of Sciences, University of Sargodha, Sargodha, Pakistan; 5 University Institute of Biochemistry & Biotechnology, PMAS Arid Agriculture University, Rawalpindi, 46300, Pakistan; * These authors contributed equally to this work

## Abstract

The identification issue of livestock can be resolved by using molecular identification tools that are acceptable to preserve and maintain pure breeds worldwide. The application of a molecular identification methodology is more important for developing nations, e.g., Pakistan, where uncontrolled crossbreeding has become a common practice and the import of exotic animals and germplasm is ever increasing. This presents a risk to local breeds as also stated by the FAO. Therefore, the current study was designed to develop standard molecular markers for Cholistani cattle to ascertain their purity for breeding purpose. In this study 50 and 48 unrelated males were sampled for Cholistani and each crossbred cattle, respectively. Candidate molecular markers present in Cholistani but absent in crossbred cattle and vice versa were detected using the amplified fragment length polymorphism (AFLP) method. Eleven markers were developed and were converted to single nucleotide polymorphism (SNP) markers for genotyping. The allele frequencies in both breeds were determined for discrimination ability using polymerase-chain-reaction–restriction-fragment-polymorphism (PCR-AFLP). The probability of identifying the Cholistani breed was 0.905 and the probability of misjudgment was 0.073 using a panel of markers. The identified markers can ascertain the breed purity and are likely to extend the facility for breed purity testing before entering into a genetic improvement program in the country.

## 1 Introduction

Pakistan possesses cattle breeds that are categorized as zebu cattle (*Bos indicus*). The local breeds can be classified as dairy, draft and dual-purpose breeds depending upon their utility either in dairying or in agricultural work. The desirable characteristics of local breeds are disease resistance, heat tolerance, ability to survive and reproduce under stress and low-input system. There are 44.4 million cattle in the country with a positive population growth rate (GoP, 2017). The share of Cholistani cattle is 1.81 % of the total population (0.804 million heads) restricted to the province of Punjab, a desert area. Almost half of the cattle population does not belong to any specific breed group and is thus categorized as nondescript. Cholistani is considered a dairy cattle breed, like the Sahiwal and Red Sindhi breeds. All of the local breeds are phenotypically characterized, and Sahiwal has maximum literature citation among dairy breeds (Afzal and Naqvi, 2004). Most of the studies on Pakistani cattle are limited to phenotypic characters while a few studies are available on genetic parameters at population level. However, environmental and underlying genetic factors do affect the accuracy of phenotypic characterization of domestic cattle. There is a scarcity of information regarding genetic characterization and breed identification among Pakistani dairy cattle breeds. There are few studies that focus mainly on genetic variation and diversity in cattle (Azam et al., 2012; Imran et al., 2012; Nasreen et al., 2012). For instance, the genetic diversity of Hariana and Hissar cattle breeds of Pakistan was investigated using 30 bovine microsatellite markers. It was concluded that although Hariana and Hissar breeds shared a common breeding tract, these breeds are genetically different enough to be identified as two separate breeds (Rehman and Khan, 2009). Azam et al. (2012) studied the diversity of Pakistani cattle breeds, viz. Tharparkar and Red Sindhi, using microsatellite markers. The above cited review of literature revealed that no work has been done to find out breed-specific markers in Pakistani dairy cattle breeds.

A number of studies were initiated to characterize the European cattle breeds in 1990s that has continued in the recent past using molecular tools like microsatellite markers (Bradley et al., 1996; Canon et al., 2001; Cooper et al., 2016; Ginja et al., 2010; Kantanen et al., 2000). A significant progress in molecular technology during last decade has made it possible to perform genetic analysis based on DNA markers. DNA markers have been used for pedigree registration, individual identification, parentage test and the removal of carrier individuals with genetic diseases among livestock species (Junqueira et al., 2017; Yudin and Voevoda, 2015).

Various molecular methods have been used to identify breeds among different farm animal species over the period of time, e.g., microsatellite markers in dogs (Koskinen, 2003), microsatellite markers in goats (Iquebal et al., 2013), microsatellite markers in cattle (Rogberg-Munoz et al., 2014), allele-specific polymerase chain reaction in chicken (Choi et al., 2007), amplified fragment length polymorphism (AFLP) in cattle (Ajmone-Marsan et al., 1997; Sasazaki et al., 2004, 2006) and single nucleotide polymorphism (SNP) chips in cattle (Cooper et al., 2014, 2016; Suekawa et al., 2010). However, AFLP markers have been extensively applied in DNA fingerprinting (Ajmone-Marsan et al., 1997; Vos et al., 1995), genetic distance analysis (Ajmone-Marsan et al., 2002), quantitative trait loci (QTL) mapping (Milanesi et al., 2008), linkage mapping (Huang et al., 2009) and lastly, of course, breed identification (Negrini et al., 2007a, b; Sasazaki et al., 2004).

It is observed that with the passage of time, breed identification methodology has been updated from simple methods, i.e., amplified fragment length polymorphism (AFLP) markers to genomic chips (Cooper et al., 2016; Gurgul et al., 2016). However, the AFLP method is still one of the cheapest techniques to provide a useful means of identification in developing countries (Milanesi et al., 2008). AFLP is also a powerful method for acquiring genome information easily because of polymorphic band detection using combinations of selective primers. Sasazaki et al. (2007) reported the usefulness of AFLP markers as a tool to discriminate between domestic and imported beef.

Cholistani cattle are very well adapted to the harsh climatic conditions of the country. It has desirable characteristics of resistance to disease and ticks, like other Pakistani cattle breeds. The breed is a favorable candidate for selection as dairy animals that can live under the low-input system of a desert climate. Hence, a traditional selection program has been carried out occasionally by the Livestock & Dairy Development Department (L&DD), Government of Punjab, under different schemes (Farooq et al., 2010). However, as a result of unrestrained extensive crossbreeding, the local, precious genetic pool of Cholistani cattle is at risk (Khan et al., 2008). The published literature review showed no work has been done to find out breed-specific markers in the Cholistani dairy cattle breed that can ascertain the purity of the breed. Therefore, the objective of the present research was to find breed-specific molecular markers for the genetic identification of Cholistani and crossbred populations to ascertain breed purity for breeding purposes.

## 2 Materials and methods

### 2.1 Animals, sample collection and DNA extraction

In this study, 50 and 48 male animals from each of the two breed populations, viz. Cholistani and crossbred cattle, were taken at random from different areas in the country following FAO guidelines on the selection animals and unrelatedness (FAO, 2011). The crossbred animals were a cross between Cholistani and Holstein Friesian with F2 or later generations. The pedigree information of sampled animals was available in the records of the Livestock Experiment Station as well as the Semen Production Unit Qadarabad and Karaniwala. Blood samples were collected in sterile tubes containing ethylenediaminetetraacetic acid (EDTA) anticoagulant and samples were shipped to the Laboratories of Animal Breeding and Genetics, PMAS Arid Agriculture University, Rawalpindi, for further analyses. Genomic DNA was extracted from blood samples according to standard manufacturer's protocols using the GeneJET Whole Blood Genomic DNA Purification Kit (Thermo Scientific). The quality of the DNA extracted was tested with Quawell Nanodrop (Q 5000, Quawell UV-VIS Spectrophotometer, USA), and DNA was kept at -20 ${}^{\circ }$C until further use in the study.

### 2.2 The AFLP method

The procedures of the AFLP method were employed as described by Vos et al. (1995). The sequence of AFLP adapters and primers is listed in Table 1. Genomic DNA (500 ng) was digested with 5 U of TaqI (Invitrogen) at 65 ${}^{\circ }$C for 1 h, followed by second digestion with 5 U of EcoRI (Invitrogen) at 37 ${}^{\circ }$C for 1 h. Double-stranded adapters were ligated to the restriction fragments, following the addition of a 5 pmol EcoRI adapter, a 50 pmol TaqI adapter, 1 mM ATP and 1 U of T4 DNA ligase at 37 ${}^{\circ }$C for 3 h. The ligated DNA fragment solution was then diluted 10-fold with 10 mM Tris–HCl (pH 8.0), 0.1 mM EDTA and stored at -20 ${}^{\circ }$C.

**Table 1:** Sequence of AFLP adapters and primers.

Name	Sequence (5${}^{\prime }$ to 3${}^{\prime }$)
EcoRI adapter	CTC GTA GAC TGC GTA CC
	AAT TGG TAC GCA GTC TAC
TaqI adapter	GAC GAT GAG TCC TGA C
	CGG TCA GGA CTC AT
EcoRI primer + 1	GAC TGC GTA CCA ATT CA
TaqI primer + 1	GAT GAG TCC TGA CCG AC
	GAT GAG TCC TGA CCG AT
EcoRI primer + 3	GAC TGC GTA CCA ATT CAN N
TaqI primer + 3	GAT GAG TCC TGA CCG ACN N
	GAT GAG TCC TGA CCG ATN N

Pre-amplification was carried out in a polymerase chain reaction (PCR) machine where adapters were used as primer. This allowed a first selection of fragments by only amplifying the DNA restriction fragments that had been ligated to adapters on both ends. Pre-amplified fragments were preselected using 75 ng each of EcoRI primer and TaqI primer with a single selective nucleotide and then reaction mixtures were diluted 10-fold with 10 mM Tris–HCl (pH 8.0) and 0.1 mM EDTA and stored at -20 ${}^{\circ }$C.

In order to restrict the level of polymorphism and to label the DNA, selective amplifications were performed using 5 ng of EcoRI primer and 30 ng of TaqI primer with three selective nucleotides. PCR products amplified with different primer combinations were loaded onto 5.0 % denaturing polyacrylamide gels, electrophoresed for 2 h and finally were detected by a SilverXpress^®^ Silver Staining Kit (Life Technologies, USA).

Afterwards, selected bands of selective amplification were excised and purified using a GeneJET Gel Extraction Kit (Thermo Scientific, USA) using the manufacturer's protocol. The extracted samples were used to carry out PCR under standard conditions with the primers used in the selective amplification of AFLP assays. The amplified PCR products were cloned in a pUCM-T Cloning Vector Kit (Bio Basic Inc., Canada) according to the manufacturer's instructions, transformed by heat shock method to DH5α and spread on media plates overnight at 37 ${}^{\circ }$C. Positive colonies were picked up and cultured overnight in a Luria–Bertani medium, to isolate the plasmids. The product size of the original DNA fragment was determined by restriction and electrophoresis. The plasmid was sequenced by Macrogen Korea.

### 2.3 Sequences analysis

All sequences were analyzed for homology to a database using the online site of the NCBI (http://www.ncbi.nlm.nih.gov/BLAST/, last access: 17 December 2017) running the BLAST programs (Gibney and Baxevanis, 2011). Genotype information of sequenced fragments was obtained from blasting two breed sequences between them and across the whole genome with an average SNP spacing of 51.5 Kb at chromosome positions. Based on the genotyping data, the allele frequency at each SNP was calculated and used to select candidate SNPs. Finally, the primers were designed on the SNP flanking site of selected SNPs. The region including the SNP was amplified using PCR methods. PCR was performed in a volume of 20 µL using DreamTaq Green PCR Master Mix (Thermo Scientific, USA). PCR was carried out using a standard PCR program with 2 min denaturation at 94 ${}^{\circ }$C, 30 cycles for 1 min at 94 ${}^{\circ }$C, 30 s annealing at temperatures, 30 s extension at 72 ${}^{\circ }$C, and a final extension for 7 min at 72 ${}^{\circ }$C. The bands were selected as markers based on the frequency in Cholistani or crossbred cattle. The selection criteria were presence at the frequency of less than 15 % in Cholistani and more than 75 % in crossbred cattle. This yielded 11 amplicons used in the current study. Thus, these amplicons were used as markers. The genotype frequencies of these makers on the subject animals were investigated in order to examine their application as breed-specific markers.

### 2.4 Statistical analysis

In order to adequately evaluate the efficiency of these markers for the discrimination between both dairy cattle breeds, the probability of identification was calculated based on the estimated allelic frequency of each marker. Analyses of Hardy–Weinberg equilibrium (HWE) and a likelihood ratio test of linkage disequilibrium, within-breed diversity of haplotypes and expected heterozygosity were performed using the program Arlequin (Excoffier and Lischer, 2010). Moreover, the probability of identification ($P_{\mathrm{is}}$) and the probability of misjudgment ($P_{\mathrm{ms}}$) of the Cholistani breed were calculated using different panels of markers following the method used by Sasazaki et al. (2004).

## 3 Results and discussion

The present research was aimed to develop specific molecular markers for the discrimination of Cholistani cattle from crossbred population. The approach used was AFLP, previously applied by Sasazaki et al. (2006) for the discrimination between Japanese black and crossbred populations.

**Table 2:** Marker information for polymerase-chain-reaction–restriction-fragment-polymorphism (PCR-AFLP).

Marker	Forward primer ($5^{\prime }\to 3^{\prime }$)	Annealing	Product	Mutations
	Reverse primer ($5^{\prime }\to 3^{\prime }$)	temperature	size	
		(${}^{\circ }$C)	(bp)	
LABG1	GAGTGTAGTTGATTTATTTTTATTTGT	65	170	6 bp
	GAGTACTGACGCAGCACACCTACAGCC			insertion/deletion
LABG2	GTAAAACAACTTAGTGGTGAATTCGGG	65	238	SNP at EcoRI
	TCGGATTGCTTACGTGCCTTTCTGGAGAC			site A → G
LABG3	CCTTTGTCTTCCACTGCCCACCTGTCA	65	155	SNP at TaqI
	CACATCTCTTTAGCACTCTCGTTCTGGT			site G → A
LABG4	TAGGGAAGATACCACAATAAGTAAAG	65	134	SNP at TaqI
	GTAAAGATAAACATGTAAAGATATAGCACAGCATCGACC			site A → G
LABG5	TGTTACAACGCAAGGCTGGGAAACTG	65	190	SNP at TaqI
	GAGAGTGGAGAGAATAGCGGATGCCTCGACCTGACTTTC			site G → T
LABG6	CGGGCTGGTCTGAGAAAAGTCAAGTCAC	65	570	1 bp
	CAGTCAATGAAGAGCCGAGTAGAAGAAC			insertion/deletion
LABG7	TCTTGGTCACCTGCTGCTTCCTGTCCTG	63	498	SNP at TaqI
	CGTATCCGTAGTATAGTAGTATGGTG			site T → C
LABG8	ATTCTATCAACAGCAAAAACCAAGCATT	63	99	1 bp
	AAATGGCAGGAAGGAAGGCTATAGATGG			insertion/deletion
LABG9	CCCAAGGTCTAAGAGCCAGGGTACTGATGC	59	127	8 bp
	TCTGTAAAGACAAAGTGAATCTCTAAGG			insertion/deletion
LABG10	ACCCCCGTCCTTCTTCCCCATCACAGCC	65	99	3 bp
	GCAGACAACAGGAAGACCCGTAAGTTTC			insertion/deletion
LABG11	CACATGATACAGCAAAAGGAGTTC	65	107	SNP T → G
	CCCAATGTTCTGACGTCTTCCGA			

### 3.1 AFLP markers

In the current study we adapted AFLP markers for breed documentation since these markers are more informative (Sasazaki et al., 2006). The comprehensive information on each of the markers used, including their forward as well as reverse primers, annealing temperatures, product size and pertinent mutation, are given in Table 2. The annealing temperature of all the 11 markers ranged from 59 to 65 ${}^{\circ }$C whereas the product sizes of given markers ranged from 99 to 570 bp. The product sizes were different from the previously reported studies (Sasazaki et al., 2004, 2006). This difference could be credited to a difference in breeds. Moreover, mutations conforming to the given markers were the result of either insertion/deletion or SNP. The size, location and relevant gene information for each of the 11 markers is mentioned in Table 3 where the location of LABG3 remained unknown.

### 3.2 Within-breed diversity of haplotypes and expected heterozygosity

Estimates of inbreeding and heterozygosity have previously been documented using AFLP markers (Dasmahapatra et al., 2008). In the current study, the within-breed diversity of haplotypes and expected heterozygosity of each marker is mentioned in Table 4. The gene diversity value was slightly higher in crossbred than Cholistani cattle (0.978±0.0004 vs. 0.981±0.0004). The genetic diversity in the current study is quite high compared to previous studies for Cholistani as well as other Pakistani cattle breeds (Hussain et al., 2016; Rehman and Khan, 2009). The difference could be attributed to the difference in markers, i.e., microsatellites in previous studies and AFLP in the current study. Moreover, the expected heterozygosity for LABG8 & 10 was higher in crossbred than Cholistani cattle, but the reverse was true in the case of LABG2 and 9 (Table 4). Moreover, in Cholistani cattle only 7 markers showed heterozygosity whereas in the crossbred population, 10 markers showed heterozygosity. The overall heterozygosity observed in the current study was lower compared to that reported previously (0.32 vs. 0.67) by Hussain et al. (2016). This difference could be because of a difference in the markers used (microsatellite vs. AFLP).

**Table 3:** Result of cattle genome BLAST (all assemblies Annotation Release 105) on each marker. BAG is BCL2 Associated Athanogene 1, and LNX is Ligand of Numb-Protein X.

Marker	Size (bp)	Score	E value	Location	Gene
LABG1	170	121	$4\times 10^{-10}$	BTA-1	Thymosin beta-4
LABG2	238	231	$2\times 10^{-108}$	BTA-14	RNA-binding Raly-
					like protein
LABG3	155	121	$2\times 10^{-10}$	Un	No corresponding gene
					found during genome
					BLAST
LABG4	134	131	$9\times 10^{-59}$	BTA-5	Ras-related and
					estrogen-regulated
					growth inhibitor
LABG5	190	306	$1\times 10^{-71}$	BTA-5	BAG family molecular
					chaperone regulator 1
LABG6	570	991	0.0	BTA-11	Spermatid perinuclear
					RNA-binding protein
LABG7	498	714	0.0	Un	Similar to PTK2
					protein
LABG8	99	66	$1\times 10^{-06}$	BTA-23	Hereditary
					hemochromatosis
					protein precursor
LABG9	127	70	$2\times 10^{-07}$	BTA-1	Golgin subfamily B
					member 1 isoform X1
LABG10	99	48	0.11	BTA-3	Chromodomain-
					helicase-DNA-binding
					protein 1-like
LABG11	107	113	$2\times 10^{-22}$	BTA-6	E3 ubiquitin protein
					ligase LNX

**Table 4:** Within-breed diversity of haplotypes and expected heterozygosity of Cholistani and crossbred cattle.

Breed	Diversity parameters			Expected heterozygosity	
Cholistani	Sum of square frequencies	0.024		Locus	Expected
					heterozygosity${}^{*}$
	Gene diversity	0.978±0.0005		LABG2	0.498
	Theta (Hom)	42.175±0.977		LABG4	0.494
	Theta (k)	10.780		LABG5	0.136
	Theta (S)	1.047±0.436	LABG6	0.215	
	Theta (Pi)	2.227±1.361		LABG8	0.465
				LABG9	0.284
				LABG10	0.136
Crossbred	Sum of square frequencies	0.0208		LABG2	0.219
	Gene diversity	0.981±0.004		LABG3	0.187
	Theta (Hom)	49.850±1.060		LABG4	0.396
	Theta (k)	12.651		LABG5	0.458
	Theta (S)	1.461±0.528		LABG6	0.117
	Theta (Pi)	2.752±1.615		LABG7	0.153
				LABG8	0.500
				LABG9	0.250
				LABG10	0.430
				LABG11	0.041

${}^{*}$ Results are only shown for polymorphic loci.

**Table 5:** Allelic frequencies of Cholistani and crossbred cattle for 11 markers.

Locus	Allelic frequency				
	Cholistani			Crossbred	
	Allele1	Allele2		Allele1	Allele2
LABG1	1.000±0.000	0.000		1.000±0.000	0.000
LABG2	0.463±0.024	0.537±0.024		0.875±0.014	0.125±0.014
LABG3	1.000±0.000	0.000		0.896±0.013	0.104±0.013
LABG4	0.561±0.023	0.439±0.023		0.729±0.019	0.271±0.019
LABG5	0.927±0.012	0.073±0.012		0.646±0.021	0.354±0.021
LABG6	0.878±0.015	0.122±0.015		0.938±0.011	0.063±0.011
LABG7	1.000±0.000	0.000		0.083±0.012	0.917±0.012
LABG8	0.366±0.023	0.634±0.023		0.521±0.022	0.479±0.022
LABG9	0.829±0.018	0.171±0.018		0.854±0.015	0.146±0.015
LABG10	0.073±0.012	0.927±0.012		0.688±0.020	0.313±0.020
LABG11	1.000±0.000	0.000		0.979±0.006	0.021±0.006

### 3.3 Allelic frequencies

Allelic frequencies were estimated as given in Table 5. The frequency of allele 1 ranged from 0.073 to 1.00 in the Cholistani population and 0.083 to 1.00 in the case of the crossbred population whereas the frequency of allele 2 ranged from 0.00 to 0.927 for Cholistani cattle and from 0.00 to 0.917 for crossbreds. The absence of the PCR band indicated allele 1 whereas the appearance of the PCR band indicated allele 2 in the current study. Therefore, allele 2 of LABG2, 4, 6, 8 and 10 was indicated as a probable breed identification marker for Cholistani and crossbred cattle. A similar methodology was previously adopted to identify Japanese black cattle and crossbred populations (Sasazaki et al., 2004, 2006). However, frequencies were different between the current and previous studies because of different populations.

### 3.4 Power of identification ($P_{\mathrm{is}}$) and misjudgment ($P_{\mathrm{ms}}$)

The Hardy–Weinberg equilibrium (HWE) was tested for each locus using genotypic results of both Cholistani and crossbred populations. None of the loci showed a significant departure from HWE at P<0.05 for the probability test in the population. The linkage disequilibrium between a pair of loci was subsequently tested using a likelihood ratio test. None of the locus pairs showed significant disequilibrium at P<0.05. Therefore, the calculations of identification and misjudgment probability described below were based on the assumption of no linkage among the 11 markers (Sasazaki et al., 2006).

The allele frequencies retrieved from genotype results are given in Table 5. Allele 2 was identified for use as breed identification marker using PCR technique to discriminate between Cholistani and crossbred populations in the country. The frequency of allele 2 ranged from 0.00 to 0.927 for all loci in Cholistani and from 0.00 to 0.917 in crossbred cattle. The loci having higher frequencies in Cholistani and lower frequencies in crossbred cattle were used for further analysis. The probabilities of identification and misjudgment were calculated following Sasazaki et al. (2004).

**Table 6:** Identification ($P_{\mathrm{is}}$) and misjudgment ($P_{\mathrm{ms}}$) probabilities of the Cholistani breed using different panels of markers.

Stage	Marker	$P_{\mathrm{is}}$	$P_{\mathrm{ms}}$
First	LABG8	0.634	0.479
	LABG6	0.122	0.063
	LABG8 + 6	0.679	0.512
Second	LABG2	0.537	0.125
	LABG4	0.439	0.271
	LABG10	0.927	0.313
	LABG2 + 4	0.236	0.034
	LABG2 + 10	0.498	0.039
	LABG4 + 10	0.407	0.085
	LABG2 + 4 + 10	0.703	0.073
Total	LABG2 + 4 + 6 + 8 + 10	0.905	0.073

The probability of judgment and misjudgment was calculated based on the frequency of markers LABG2, 4 6, 8 and 10 in Cholistani and crossbred populations. The highest probability was observed in LABG10 (0.927) with the probability of misjudgment being 0.31 followed by LABG2 and 4 (Table 6). Sasazaki et al. (2004) reported the highest probability of 0.620 for a single allele in his study of Japanese black cattle. The difference could probably be attributed to the different architecture of the breeds involved in studies. For instance, Sasazaki et al. (2004) had both *Bos taurus* breeds whereas we had genotypes of *Bos Taurus* (crossbred) as well as *Bos indicus* (Cholistani). A panel of markers containing LABG2, 4 and 10 showed an improved probability of identification (0.702) with the probability of misjudgment being 0.073. Furthermore, the combined probability of identification for LABG2, 4, 6, 8 and 10 exhibited further improvement as 0.905. Overall, results were comparable to earlier studies (Sasazaki et al., 2004, 2006). However, the probability of misjudgment was higher in the current study as compared to previous reports (Sasazaki et al., 2004, 2006). This difference could again be because of the breed difference. Finally, the use of markers developed from the present study with larger number of samples might improve the accuracy.

## 4 Conclusions

The current study generated molecular breed-specific markers to identify the purity of the Cholistani breed. The results showed that the Cholistani breed can be tested for purity before entering into a breeding program where the probability for judgment and misjudgment was 0.905 and 0.073, respectively. The relatively higher probability of misjudgment might need further analysis of these markers with a larger number of samples. Moreover, the use of an SNP panel of markers for identification could be another alternative.

## Supplement

10.5194/aab-61-387-2018-supplement The supplement related to this article is available online at: https://doi.org/10.5194/aab-61-387-2018-supplement.

## Data Availability

Data access may be requested from corresponding author.
