# Supplementary material for: Development of amplified fragment length polymorphism (AFLP) markers for the identification of Cholistani cattle
Source: Arch Anim Breed. 2018 Oct 12;61(4):387–94. doi: 10.5194/aab-61-387-2018 (PMC7065403; doi:10.5194/aab-61-387-2018)

Supplement of Arch. Anim. Breed., 61, 387–394, 2018
https://doi.org/10.5194/aab-61-387-2018-supplement
© Author(s) 2018. This work is distributed under
the Creative Commons Attribution 4.0 License.

Open Access
Archives Animal Breeding

Supplement of

Development of amplified fragment length polymorphism (AFLP) markers for the identification of Cholistani cattle

Muhammad Haseeb Malik et al.

Correspondence to: Muhammad Moaeen-ud-Din (drmoinawan@gmail.com)

The copyright of individual parts of the supplement might differ from the CC BY 4.0 License.

Supplementary file

Samples collection

Samples collection from BLPRI Khairimurat and field

Figures: Samples collection from BLPRI Khairimurat and field

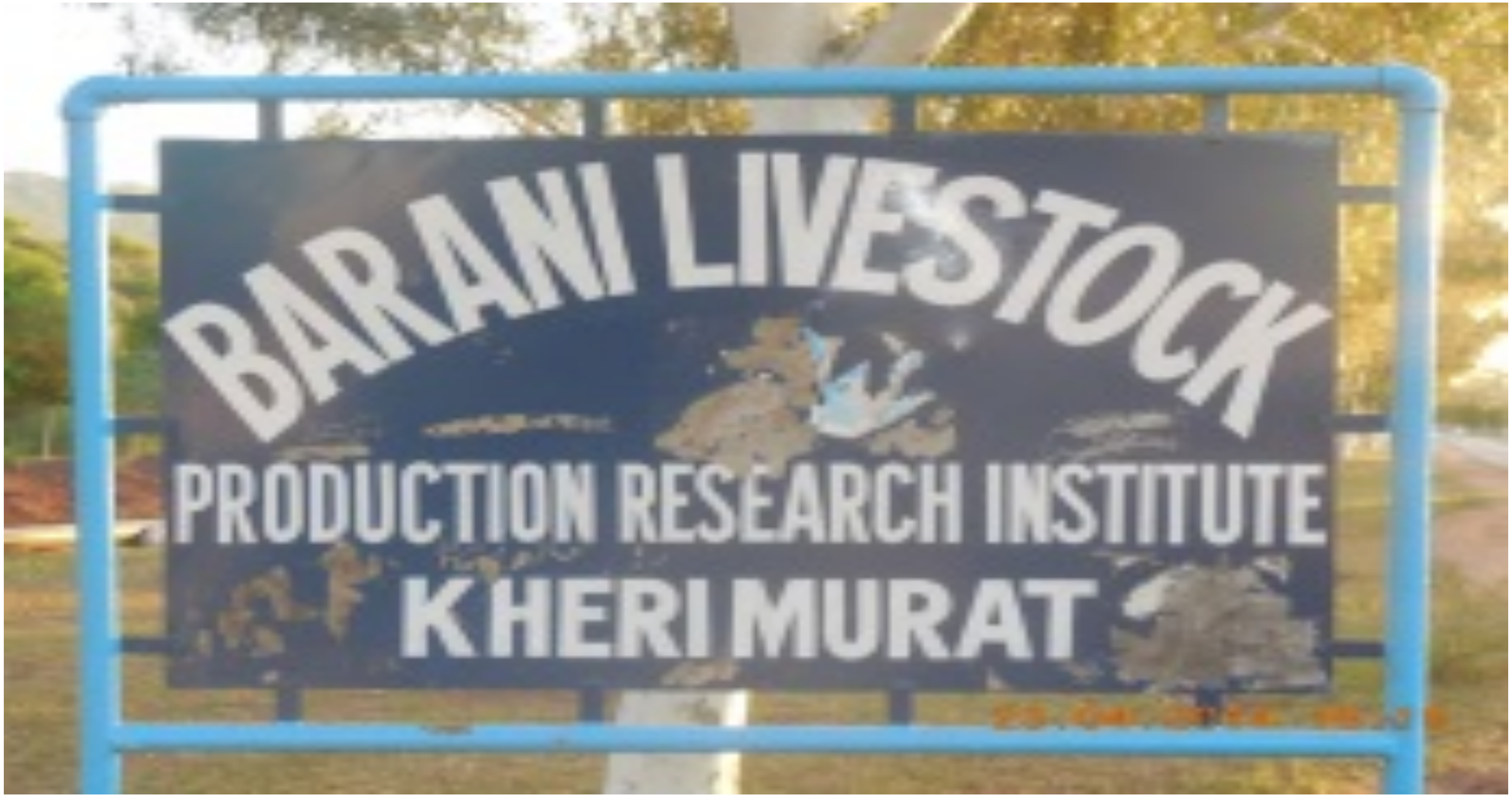

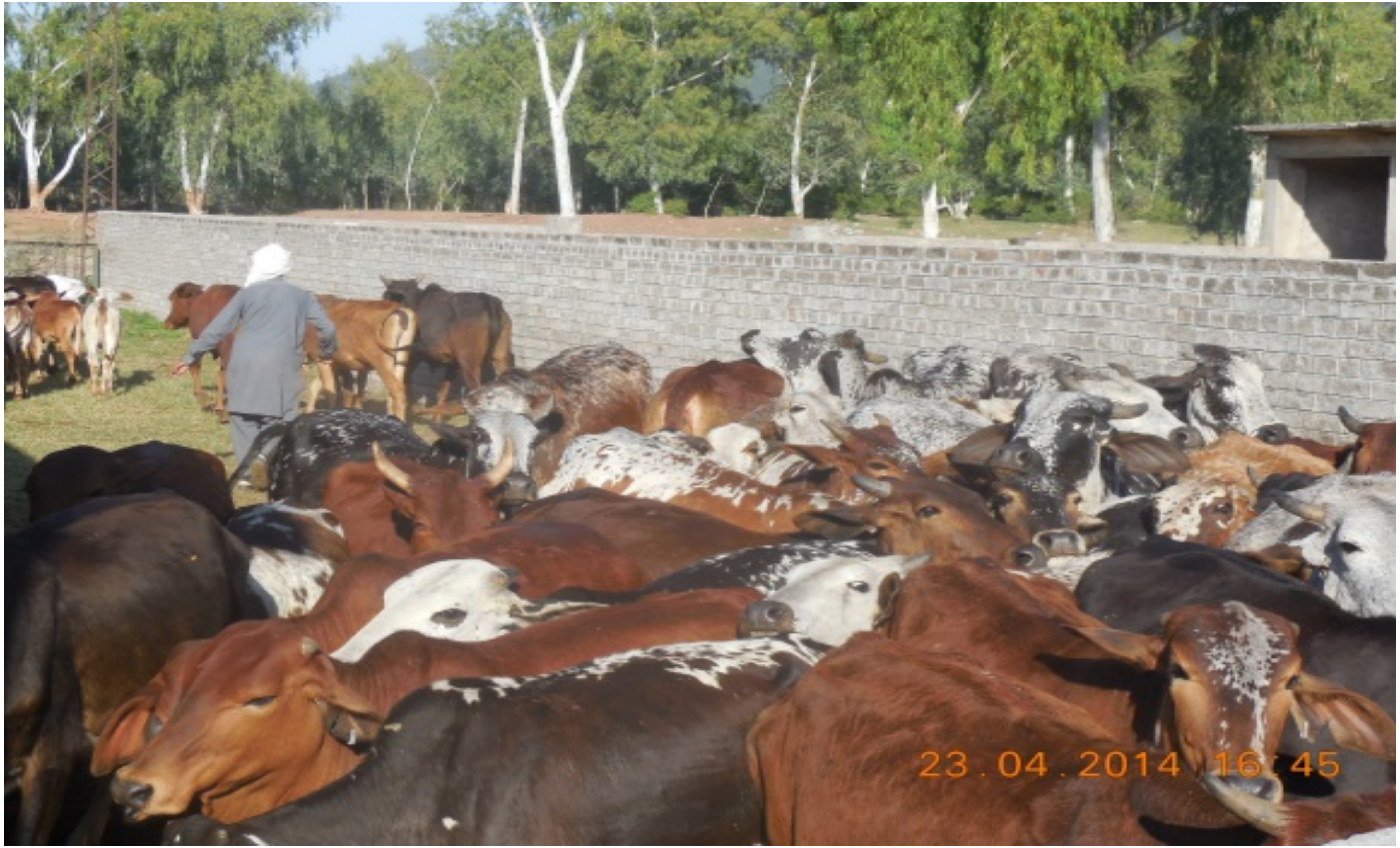
23.04.2014 16:45

Cholistani

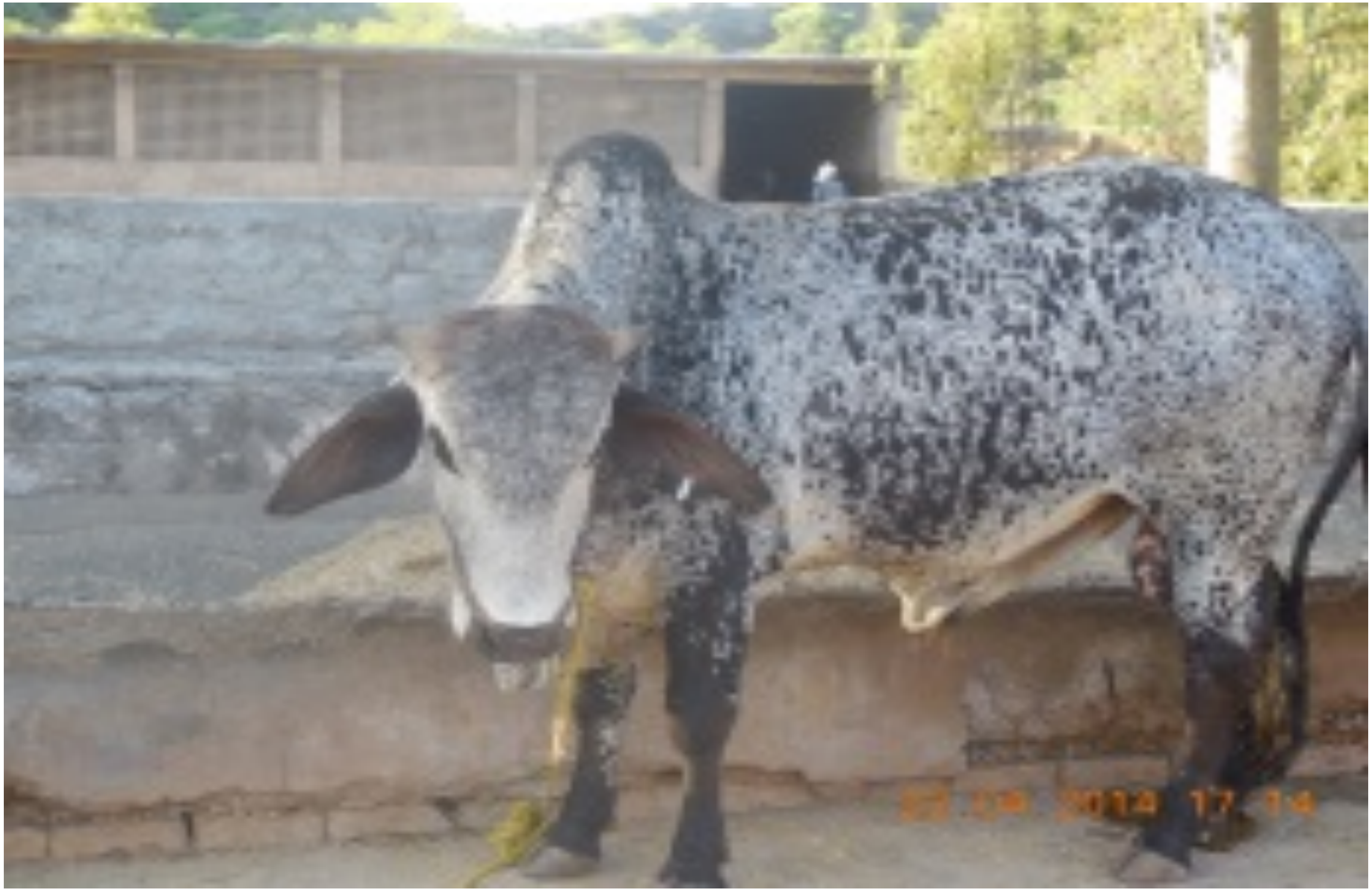

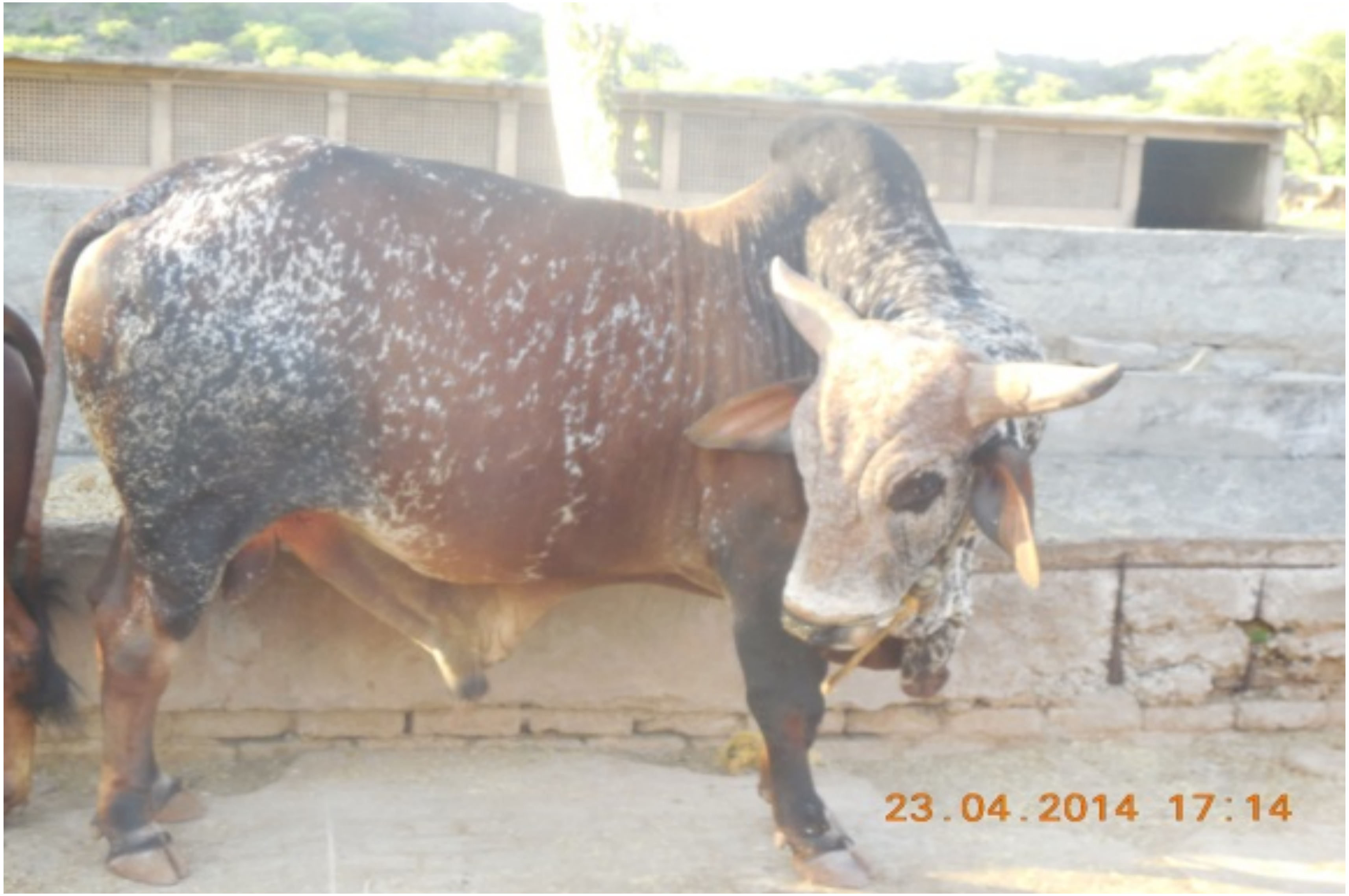
23.04.2014 17:14

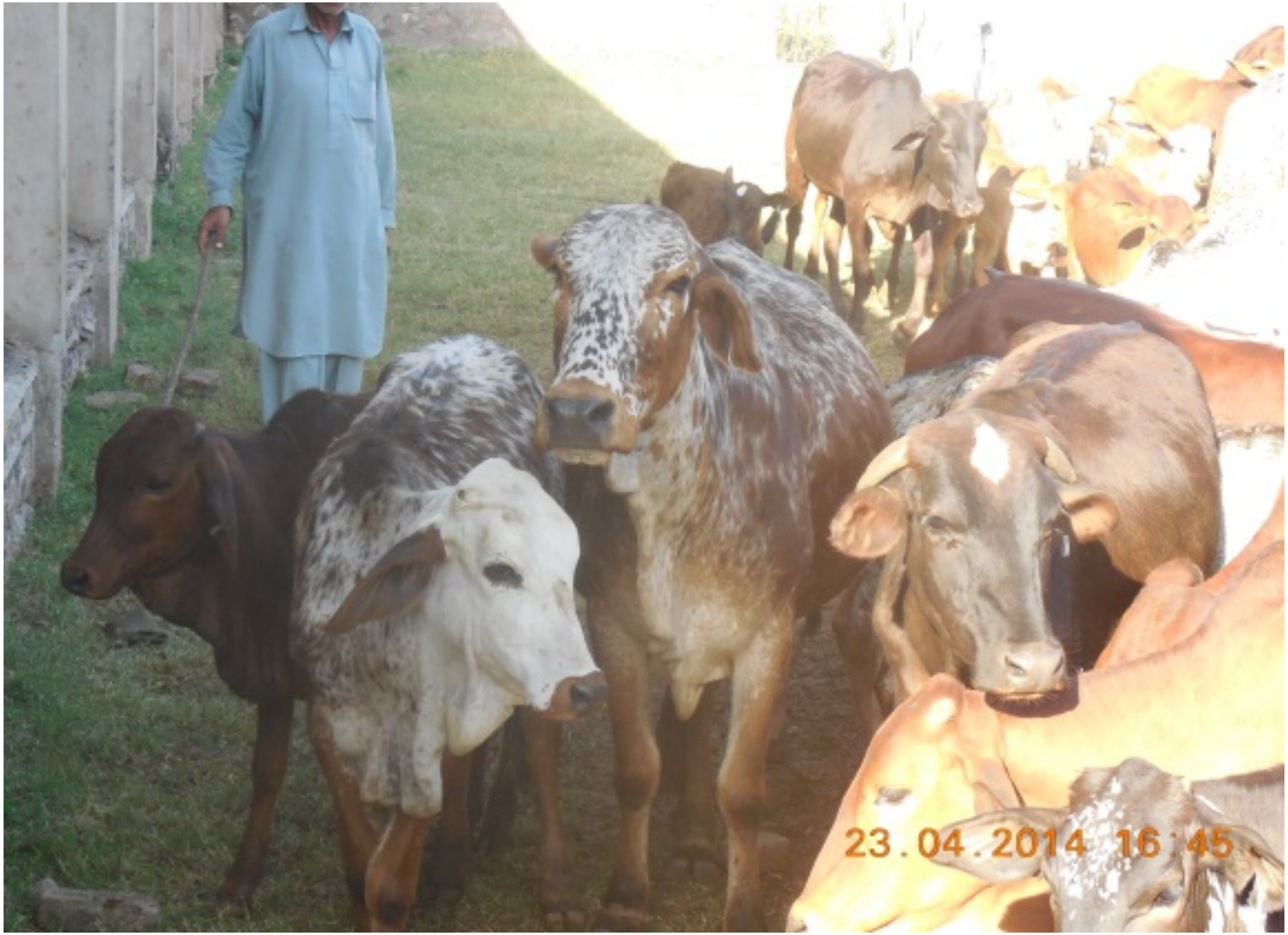
23.04.2014 16:45

Samples collection from SPU Qadarabad, SPU Karaniwala, LES Jugeetpir and field

Figure 1: Samples collection from SPU Qadarabad, SPU Karaniwala, LES Jugeetpir and field

Cholistani

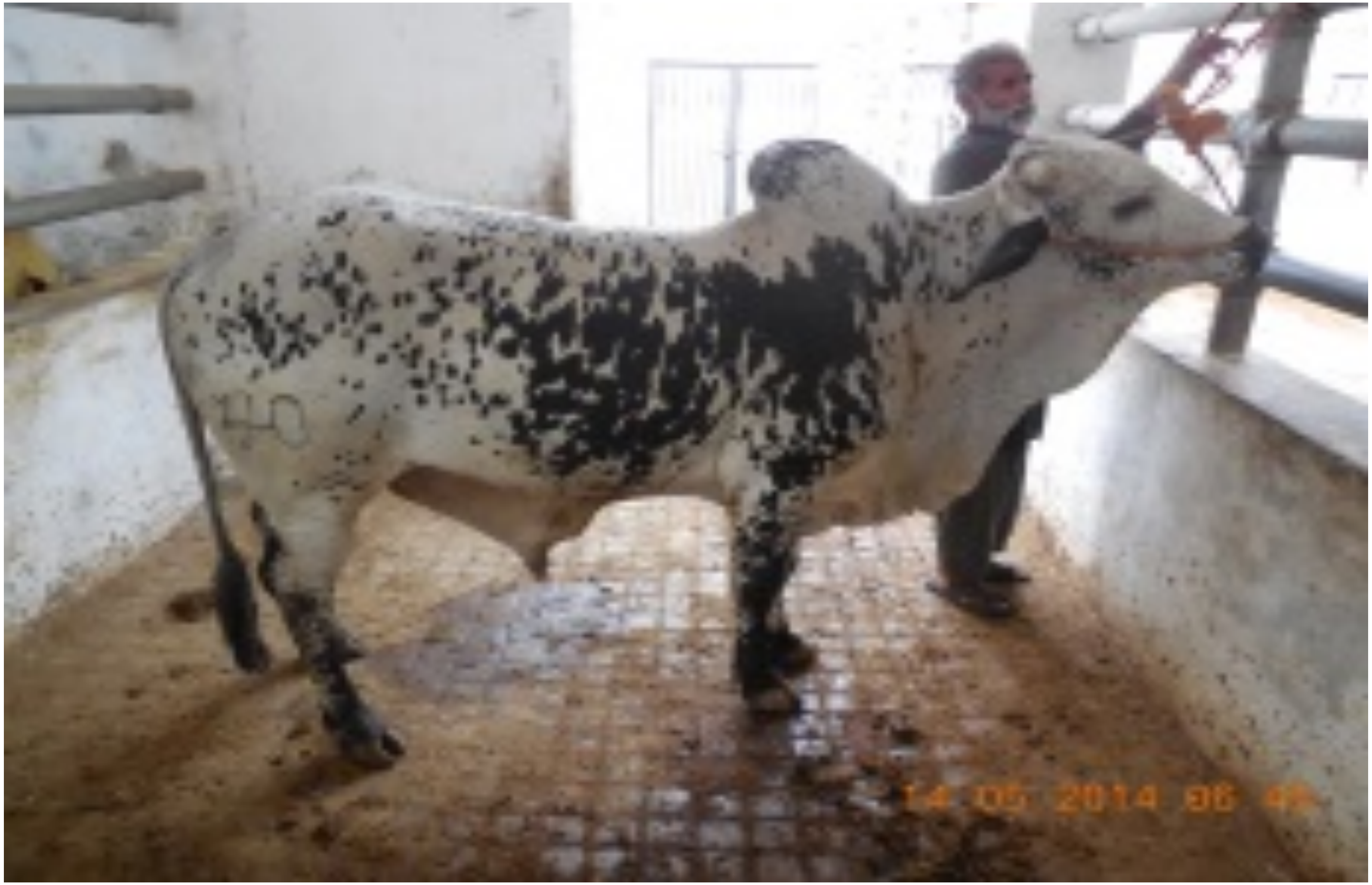

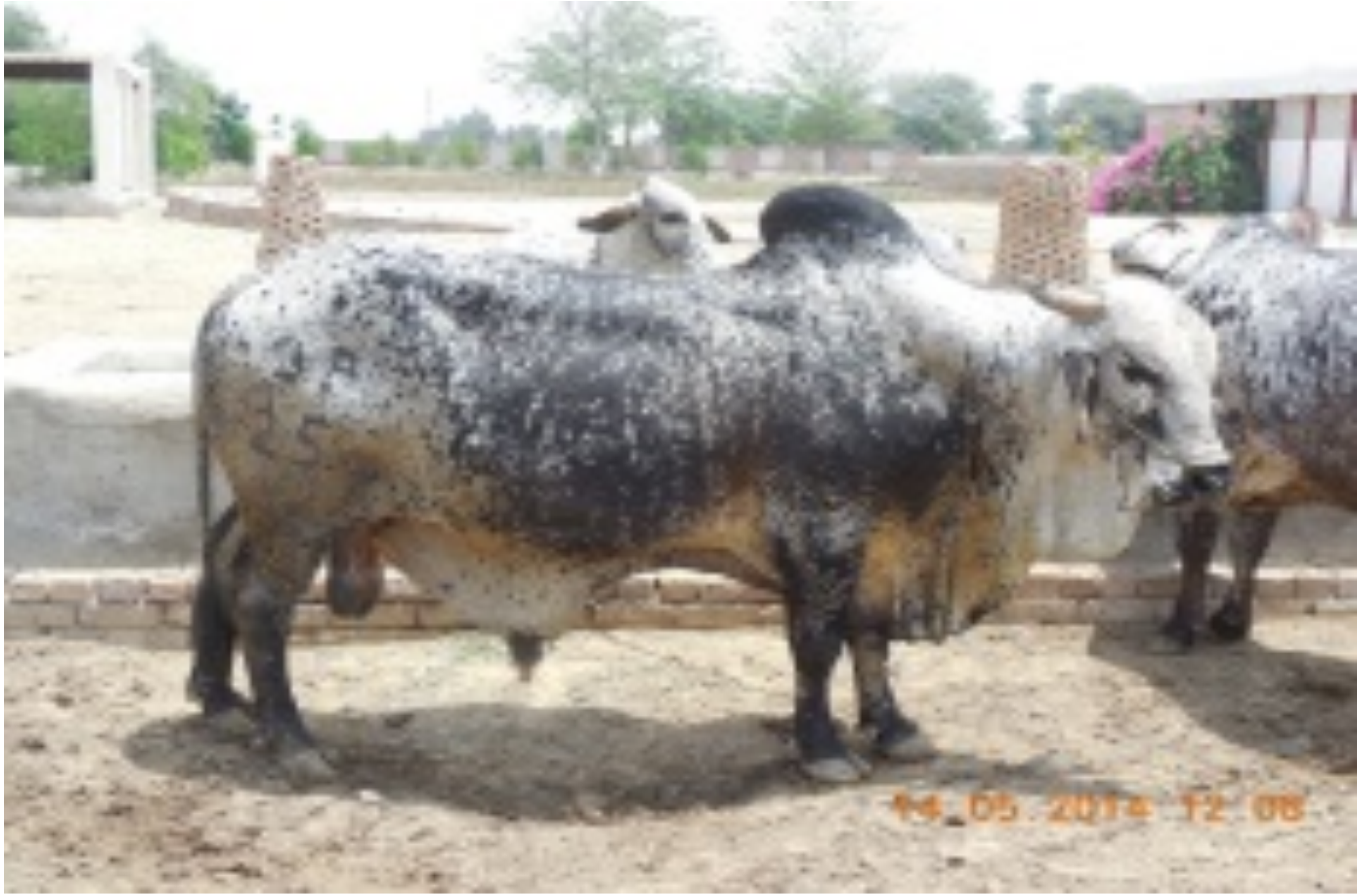

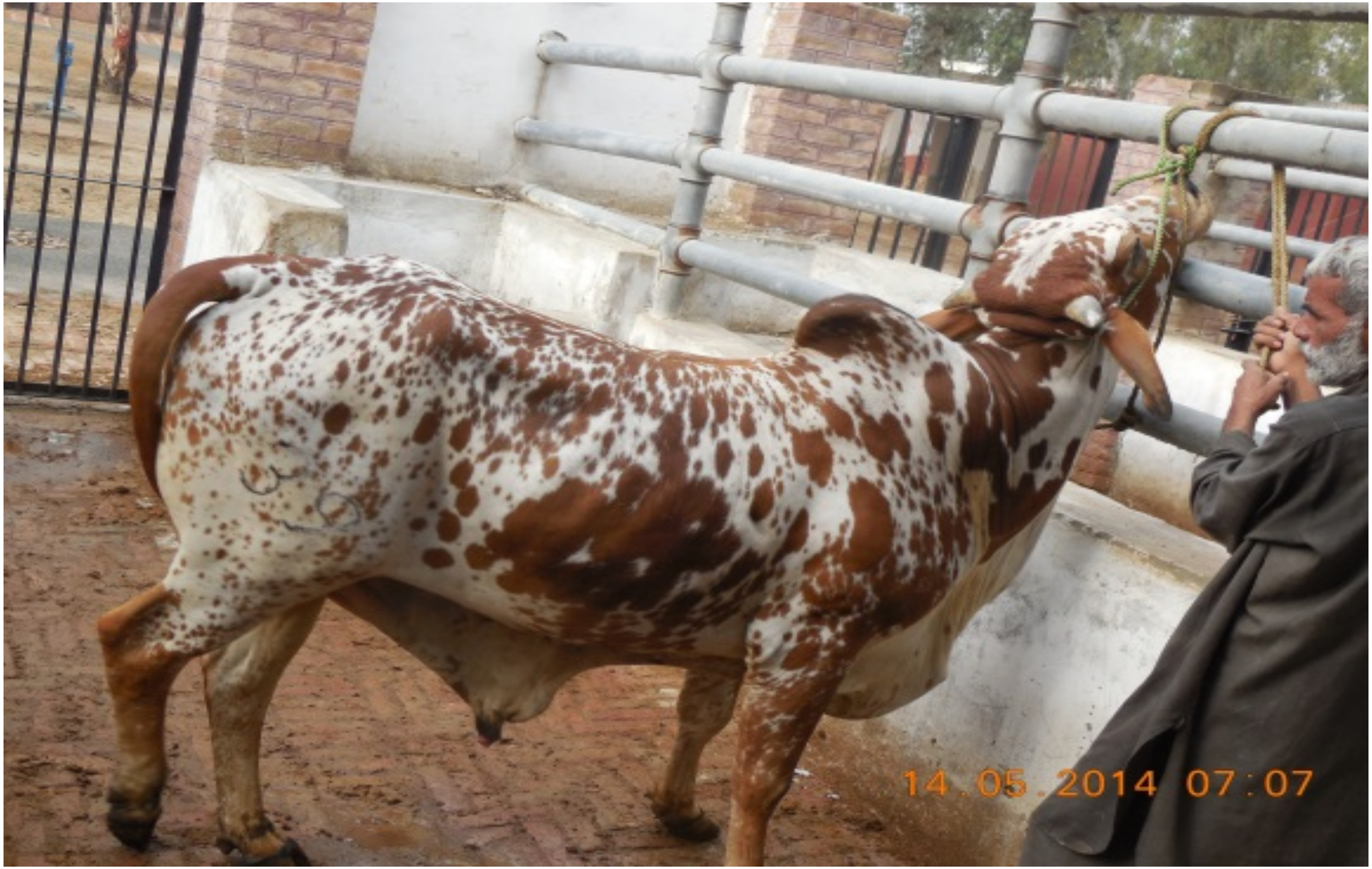
14.05.2014 07:07

Figure: DNA extracted from sampled animals

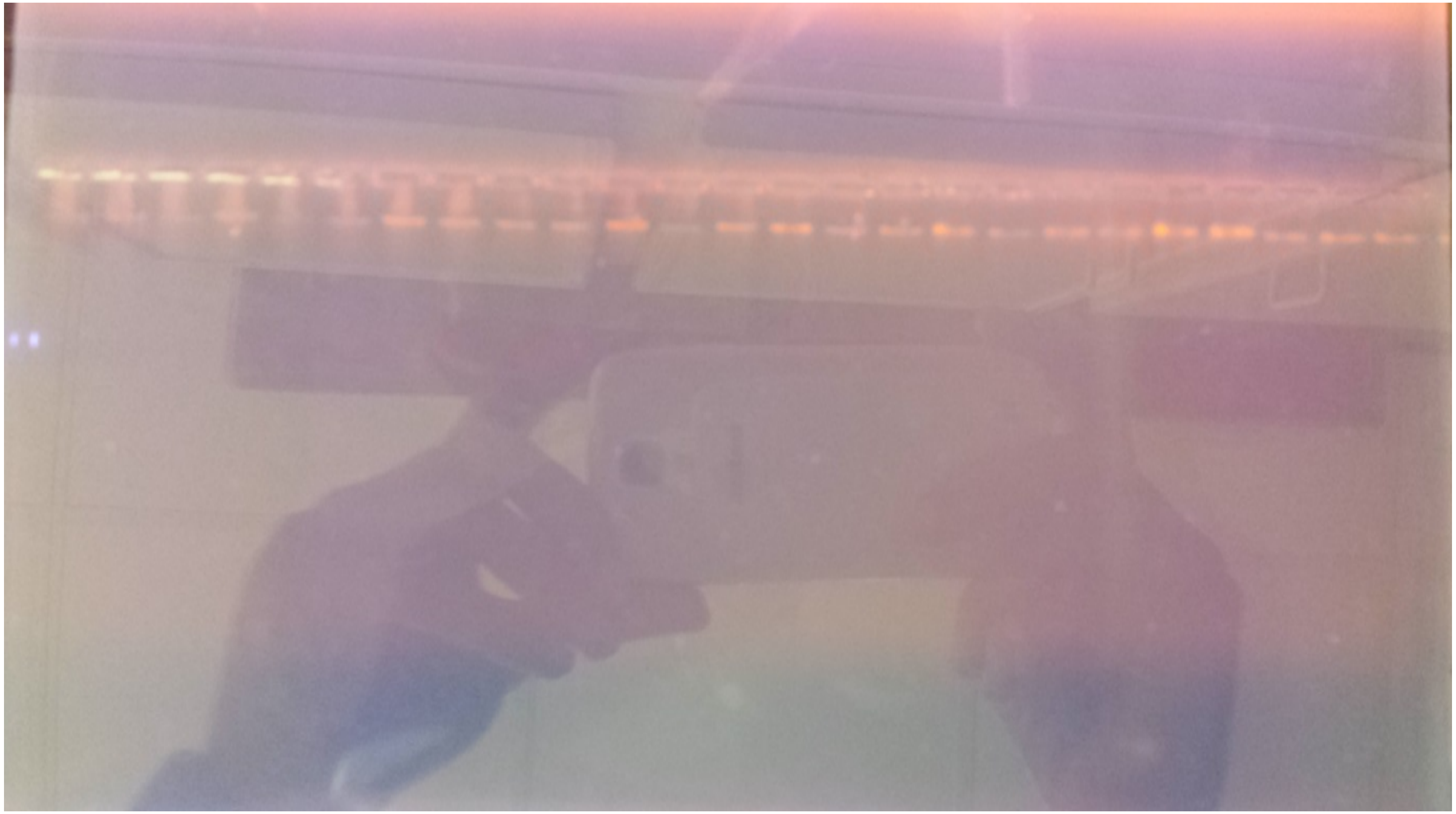

Supplement: The supplement related to this article is available online at: https://doi.org/10.5194/aab-61-387-2018-supplement. [file aab-61-387-supplement.pdf]
